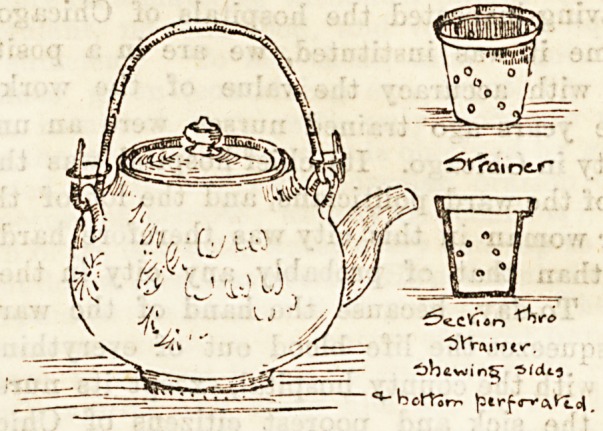# Hygienic Teapots

**Published:** 1893-07-08

**Authors:** 


### PRACTICAL DEPARTMENTS.

### HYGIENIC TEAPOTS.

We have often felt surprise that these delightful teapots are not more generally used than is the case. The little drawing we give shows at a glance the method by which the tea is made. The proper quantity of tea leaves is put into the little strainer (of which we give a section), which fits into the teapot, the boiling water poured through, and if this is done slowly it will be found that in less than three minutes the tea is of the right strength, and the superiority of flavour will not be denied by anyone who has given the plan a trial. In purchasing these teapots it should be seen to that the lid will fit the teapot itself when the strainer has been removed, for it will often be found convenient, when it is a question of keeping a fresh cup for some late comer, to take away the strainer altogether, when by the aid of the now rather despised cosy, the tea can be kept hot for a considerable time, without acquiring the bitter flavour of the tannin which makes it at once unwholesome and nasty. A great deal is heard about the injurious effects of much tea drinking, and we are afraid there is a good deal of truth in the accusations against nurses in this respect. It is a great temptation to a weary woman to fly to the teapot, for tea has undoubtedly an exhilarating effect, but when, as is so often the case, it has been what in domestic phrase is called "drawing" for a certain time, it is little short of poisonous. Fresh tea is generally admitted to be a harmless beverage, but five minutes is the longest time that the leaves should be allowed to remain.

It is well known amongst lovers of the teapot that the common earthenware ones make really the best tea, and these may now be had in this improved form, and as they are very little, if at all, dearer than the ordinary kind, they are within the reach of everyone in the matter of price. Very pretty Japanese teapots can also be had, fitted with these strainers, and for the modest sum of 1s., at Liberty's. We hope our readers will not only try the Hygienic teapots for themselves, but will also recommend them on all sides and amongst their friends.

## Figures and Tables

**Figure f1:**